# Use of a 12 months' self-referral reminder to facilitate uptake of bowel scope (flexible sigmoidoscopy) screening in previous non-responders: a London-based feasibility study

**DOI:** 10.1038/bjc.2016.43

**Published:** 2016-03-15

**Authors:** Robert S Kerrison, Lesley M McGregor, Sarah Marshall, John Isitt, Nicholas Counsell, Jane Wardle, Christian von Wagner

**Affiliations:** 1 Health Behaviour Research Centre, Department of Epidemiology and Public Health, University College London, 1-19 Torrington Place, London WC1E 7HB, UK; 2 St. Mark's Bowel Cancer Screening Centre, St. Mark's Hospital, Watford Road, Harrow, Middlesex HA1 3UJ, UK; 3 Resonant Behaviour Change and Social Marketing, Canterbury Court, 1-3 Brixton Road, London, SW9 6DE, UK

**Keywords:** colorectal cancer screening, flexible sigmoidoscopy screening, reminders, patient education, leaflet

## Abstract

**Background:**

In March 2013, NHS England extended its national Bowel Cancer Screening Programme to include ‘one-off' Flexible Sigmoidoscopy screening (NHS Bowel Scope Screening, BSS) for men and women aged 55. With less than one in two people currently taking up the screening test offer, there is a strong public health mandate to develop system-friendly interventions to increase uptake while the programme is rolling out. This study aimed to assess the feasibility of sending a reminder to previous BSS non-responders, 12 months after the initial invitation, with consideration for its potential impact on uptake.

**Method:**

This study was conducted in the ethnically diverse London Boroughs of Brent and Harrow, where uptake is below the national average. Between September and November 2014, 160 previous non-responders were randomly selected to receive a reminder of the opportunity to self-refer 12 months after their initial invitation. The reminder included instructions on how to book an appointment, and provided options for the time and day of the appointment and the gender of the endoscopist performing the test. To address barriers to screening, the reminder was sent with a brief locally tailored information leaflet designed specifically for this study. Participants not responding within 4 weeks were sent a follow-up reminder, after which there was no further intervention. Self-referral rates were measured 8 weeks after the delivery of the follow-up reminder and accepted as final.

**Results:**

Of the 155 participants who received the 12 months' reminder (returned to sender, *n*=5), 30 (19.4%) self-referred for an appointment, of which 24 (15.5%) attended and were successfully screened. Attendance rates differed by gender, with significantly more women attending an appointment than men (20.7% *vs* 8.8%, respectively; OR=2.73, 95% CI=1.02–7.35, *P*=0.05), but not by area (Brent *vs* Harrow) or area-level deprivation. Of the 30 people who self-referred for an appointment, 27 (90%) indicated a preference for a same-sex practitioner, whereas three (10%) gave no preference. Preference for a same-sex practitioner was higher among women than men (*χ*^*2*^=7.78, *P*<0.05), with only 67% of men (six of nine) requesting a same-sex practitioner, compared with 100% of women (*n*=21).

**Conclusions:**

Sending previous non-responders a 12 months' reminder letter with a brief information leaflet is a feasible and efficacious intervention, which merits further investigation in a randomised controlled trial.

Colorectal cancer (CRC) is the fourth most common cancer in the United Kingdom and the second leading cause of cancer deaths (Cancer Research UK, 2015a, 2015b). Screening can improve clinical outcomes by detecting cases early, when they are easier to treat (Logan *et al*, 2012; Cancer Research UK, 2014; Cancer Research UK, 2015c. In addition, screening can prevent cases through the timely detection and removal of adenomas (the precancerous lesions from which most CRCs develop) (Leslie *et al*, 2002), improving outcomes even further (Atkin *et al*, 2010; Whyte *et al*, 2012).

A recent appraisal of the options for CRC screening in England highlighted that a combination of ‘once-only' flexible sigmoidoscopy (FS) at the age of 55, delivered in conjunction with the current biennial faecal occult blood-testing strategy offered to 60–74-year olds, would be highly cost-effective, and would provide significant reductions the incidence, mortality and treatment costs associated with the disease (Whyte *et al*, 2012). As a result, NHS England subsequently introduced ‘once-only' FS screening for 55-year olds (known as Bowel Scope Screening, BSS) to the National Bowel Cancer Screening Programme in March 2013 (Department of Health, 2012).

As with all screening programmes, the extent to which the cost and public health benefits of including BSS are realised will depend largely on uptake (Whyte *et al*, 2012; Geurts *et al*, 2015). In a London-based pilot study, uptake of FS screening among the invited population was 50% (Robb *et al*, 2010); if representative of attendance in the national programme, it is expected that the addition of FS would result in an additional 2000 CRC deaths and 10 000 CRC cases being prevented in England by mid-2030 (Geurts *et al*, 2015). However, a recent analysis of uptake over the first 14 months of the programmes introduction revealed that only 43% of the invited population attended a BSS appointment, and thereby currently falls shy of the 50% benchmark (McGregor *et al*, 2015a).

Commonly cited reasons for not attending a FS appointment include a lack of current health problems; practical barriers (i.e., inconvenient appointment time, difficulties travelling to the appointment and so on); worry about pain, discomfort, or injury associated with the examination; and not wanting to know about any health issues (e.g., Vernon, 1997). For women specifically, the gender of the practitioner performing the test has also been reported as a potential barrier (Menees *et al*, 2005). In contrast to the FOBt-screening programme, women have been found to be less likely than men to have a FS (McGregor *et al*, 2015a), and the anticipated gender of the endoscopist is considered to be a contributing factor (Robb *et al*, 2010). Women have been found to be more willing to have FS if the endoscopist is female (Farraye *et al*, 2004) and are willing to wait longer for the test to ensure this happens (Varadarajulu *et al*, 2002).

Similar to other screening programmes, the BSS programme structure incorporates specific strategies to maximise uptake (i.e., pre-notification letters, reminder letters and so on) (Baron *et al*, 2008; Libby *et al*, 2011), but the test itself is unique in that it is a ‘one-off' procedure. Other screening programmes in England invite people to be screened every few years (e.g., FOBt every 2 years, mammography every 3 years), allowing additional opportunities for people to take part (Steele *et al*, 2010; Lo *et al*, 2014). In BSS, all adults are eligible to self-refer up to the age of 60, offering a similar window of opportunity for those who do not take up the initial invitation; however, there is no direct ‘reminder' of this possibility during the 5-year time frame.

This study, therefore aimed to examine the potential of a mailed 12 months' self-referral reminder that addressed key barriers by including an opportunity to choose the day and time of the appointment and the gender of the endoscopist performing the screening procedure. Because barriers to FS screening are unlikely to exist in isolation (Jones *et al*, 2010), and combining interventions that target CRC screening at a number of levels yield greater results than they do if used individually (Hewitson *et al*, 2011), additional barriers (e.g., anticipated pain) were addressed with the inclusion of a FS information leaflet, designed by a social marketing company, with a focus on engaging individuals with low literacy using social cognitive approaches to address misconceptions about screening (Bandura, 2004).

This study set out to test whether this strategy could be implemented in a centre with below average uptake and, if so, whether it met a minimum level of efficacy that would merit further investigation in a randomised controlled trial (RCT). A review of the methods and invitation process employed was included in order to help refine the strategy in any subsequent RCT.

## Materials and methods

### Study design and trial setting

We performed a single-centre feasibility study at St. Mark's Hospital in London. St. Mark's Hospital serves an ethnically diverse population with a high level of socioeconomic deprivation; the majority (56%) of people invited to take part in screening at St. Mark's during the first 14 months were living within either the most deprived areas in England (calculated using census data for individual postcodes), or the most ethnically diverse areas (74% also calculated using census data for individual postcodes) and, in total, only 40.5% of invitees attended an appointment, which was significantly less than the national average (43.5%) (McGregor *et al*, 2015a).

### Study population

Eligible participants were men and women registered with a General Practice in the London Boroughs of Brent and Harrow who had not attended BSS within one year of their initial invitation. Responders who had initially confirmed an appointment but failed to attend were excluded from the study.

### Sample size

To explore the feasibility of including a 12 months' reminder in the routine operations of the centre, we set out to run the study for a minimum of 10 weeks, with 16 previous non-responders sent a reminder per week, giving a total sample size of 160 participants. The sample size also allowed us to test for a minimum level of efficacy within the intervention arm, to check whether investigation in a RCT was merited (minimum number of self-referred appointments needed=3), as well as obtain estimates to inform the statistical parameters required to design such a study (which would be powered to confirm the efficacy of the intervention via a direct comparison with a control arm). The A'Hern single-stage design, which is commonly used in smaller studies with such aims (A'hern, 2001), confirmed that our sample (*n*=160) gave us acceptable levels for power (*β*>0.8) and significance (*α*<0.05) to test for a minimum level of efficacy, with the unacceptable response rate based on the self-referral rate of non-responders of the previous year (i.e., 0.35%), and the desired response rate based on a 5% improvement (i.e., 5.35%).

### Procedures

Eligible participants (*n*=844) were identified on the National Bowel Cancer Screening System (BCSS): an electronic system which provides an up-to-date record of individual-uptake data for patients enrolled in the national screening programme. For a period of 10 weeks (September 2014 to November 2014), 16 people per week were randomly selected for inclusion in the study from a variable weekly total of non-responders using simple computerised pseudo-random selection methods (Babbie, 2011).

**Figure 1:**
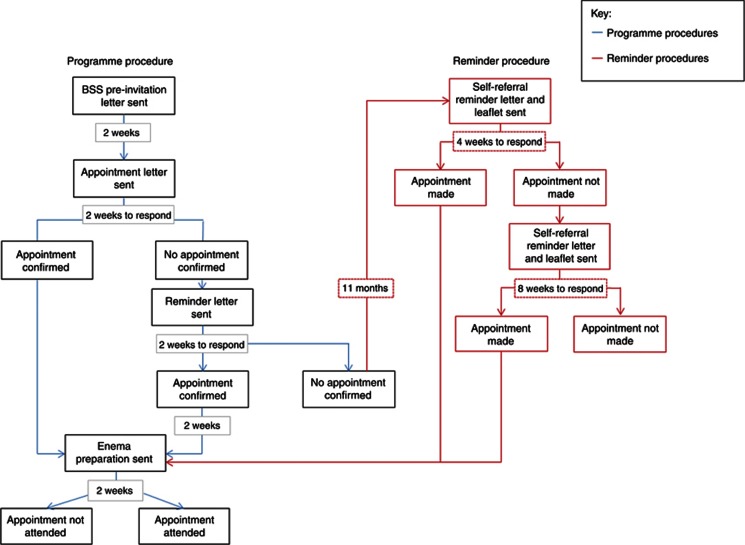
BSS invitation flowchart with self-referral reminder added.

Participants were mailed a ‘self-referral reminder letter', along with an ‘appointment–request slip', information leaflet and freepost return envelope ∼12 months after their initial invitation (12 months reminder) (see Figure 1). Participants could book an appointment either by returning their appointment–request slip, thereby initiating a call from a member of the administrative team at the centre to confirm an appointment date, or by calling the St. Mark's Bowel Cancer Screening Centre directly on the Freephone telephone number provided. Participants were able to indicate their preferences for the gender of the endoscopist and the day and time of the appointment, either on the appointment–request slip or during the phone call to the Freephone number. Participants not responding within 4 weeks of the 12 months' reminder were sent a second reminder (henceforth referred to as the ‘follow-up reminder'), which again included an appointment–request slip, the information leaflet and a freepost return envelope. After the follow-up reminder, no further attempt to contact non-responding participants was made. Participants were given a total of 12 weeks (from receipt of the 12 months' reminder) to respond before their ‘episode' was closed: any self-referrals made after this time were not included in the study results.

For those participants who made an appointment, a confirmation letter and BSS consent form was sent to their home address. The BSS consent form and confirmation letter used in this study were the same as those used for routine appointments in the national screening programme. Participants were asked to read the BSS consent form, which contained information regarding the risks and benefits of the procedure, before attending their appointment, and to call the screening centre if they had any questions. Participants were asked to bring the BSS consent form to their appointment, where a specialist screening practitioner/endoscopy nurse would discuss the risks of the procedure with the individual to ensure an informed decision to be screened was made.

The study was approved by the South Central Oxford B Research Ethics Service (Ref: 14/SC/1246).

### Intervention development

The materials used in this study were designed in conjunction with ‘Resonant': a social marketing company which specialises in the development of health behaviour change interventions (Resonant, 2015). The initial content of the leaflet and reminder letter was informed by work conducted by the UCL Research Team, which included a review of the literature on patient-specific factors for non-attendance (e.g., Vernon, 1997; Jones *et al*, 2010), and semi-structured telephone interviews with people who had recently taken part in the programme (*n*=5; three female, two male). The semi-structured telephone interviews were conducted with previously screened adults to learn more about the key factors which influenced the decision to be screened. Statements from the interviews were then selected for use in the leaflet, with permission from interviewees. Initial designs of the reminder letter and leaflet were developed by Resonant and then tested in a co-design workshop, facilitated by the company, in which screening eligible adults from the London Boroughs of Brent and Harrow (*n*=4; three male, one female; ages 55–58) gave feedback to inform future iterations. A revised version was then presented to individuals who were either the eligible age or approaching the eligible age for screening (*n*=20; 12 female, 8 male, aged 50–59 years) and feedback obtained through interviews conducted by a member of the UCL Research Team. The final leaflet (see Supplementary Appendix 1) had a Flesch readability score of 68.7, indicating that it was suitable for use within the general population (easily understood by 13–15-year olds) (Kincaid *et al*, 1975).

### 12 months' reminder letter

The 12 months' reminder letter was a personally addressed letter from St. Mark's Hospital, which: (1) invited participants to make a screening appointment by returning an ‘appointment–request slip' or by calling the Freephone telephone number for St. Mark's Bowel Cancer Screening Centre; (2) reminded participants that they had previously been invited for an appointment 1 year earlier and were eligible to make an appointment up until their 60th birthday; (3) gave participants the opportunity to select a preference for the day and time of the appointment and the gender of the practitioner performing the test and; (4) highlighted three key messages: i) that the risk of developing bowel cancer is highest in the patients' age group (55+ years), ii) screening is for people who do not have any signs or symptoms of bowel cancer and iii) screening can help prevent bowel cancer by removing small asymptomatic growths (called polyps), which have the potential to become malignant over time (see Supplementary Appendix 2).

### Leaflet

The development of the leaflet was guided by two psychological models of health behaviour that have previously been used to explain individual level factors associated with screening (e.g., perceived barriers and benefits) (Kiviniemi *et al*, 2011): the Health Belief Model (Rosenstock, 1974) and Social Cognitive Theory (Bandura, 2004). In addition, the leaflet was tailored to the London areas served by St. Mark's hospital (i.e., included a map with information about local transport links to the hospital). The leaflet also included an educational/knowledge-building component to reinforce messages regarding the benefits of screening (effectiveness and rationale), a descriptive social norms message outlining uptake of BSS at St. Mark's Hospital (‘270 people screened every month'), and several practical components designed to improve self-efficacy (i.e., instructions on how to book an appointment and directions to the hospital). In addition, factors previously found to increase screening intentions and participation were incorporated into the design, for example, male/female patient narratives (Jensen *et al*, 2014; McGregor *et al*, 2015b) (See Table 1).

### Follow-up reminder

A follow-up reminder letter was sent to individuals not responding to the 12 months' reminder within 4 weeks. This follow-up reminder repeated the information included in the 12 months' reminder, but also highlighted individuals had recently received a reminder letter (see Supplementary Appendix 3).

### Measures

Routinely available data stored on the BCSS were used to verify self-referral and attendance 4 weeks following the distribution of the 12 months' reminder, and 8 weeks following the distribution of the follow-up reminder. The BCSS was also consulted to obtain the gender, area (Harrow or Brent) and an area-based socioeconomic deprivation score for each participant. Ethnicity is not routinely collected or available on the BCSS, and so this information could not be extracted.

Socioeconomic deprivation was obtained by converting each individual's postcode to a score on the 2010 Index of Multiple Deprivation (IMD) (Department for Communities and Local Government, 2010). The IMD scores obtained were categorised into tertiles of their national distributions. The IMD uses census-derived indicators of income, education, employment, environment, health and disability, barriers to housing and services, and crime at small-area level to generate a scale ranging from 0 (least deprived) to 80 (most deprived) (Department for Communities and Local Government, 2010).

### Analysis

Descriptive statistics were used to test whether the number of self-referred appointments exceeded the threshold for further investigation in a RCT. To explore possible variations of the impact of the intervention in relation to deprivation, area and gender, a multivariate logistic regression analysis was performed (Engel, 1988). Differences in patient preferences for a same-sex practitioner were examined by gender using the *X*^2^-test of independence (Pearson, 1900); the data were analysed using SPSS Statistics (version 22).

## Results

### Sample characteristics

**Figure 2:**
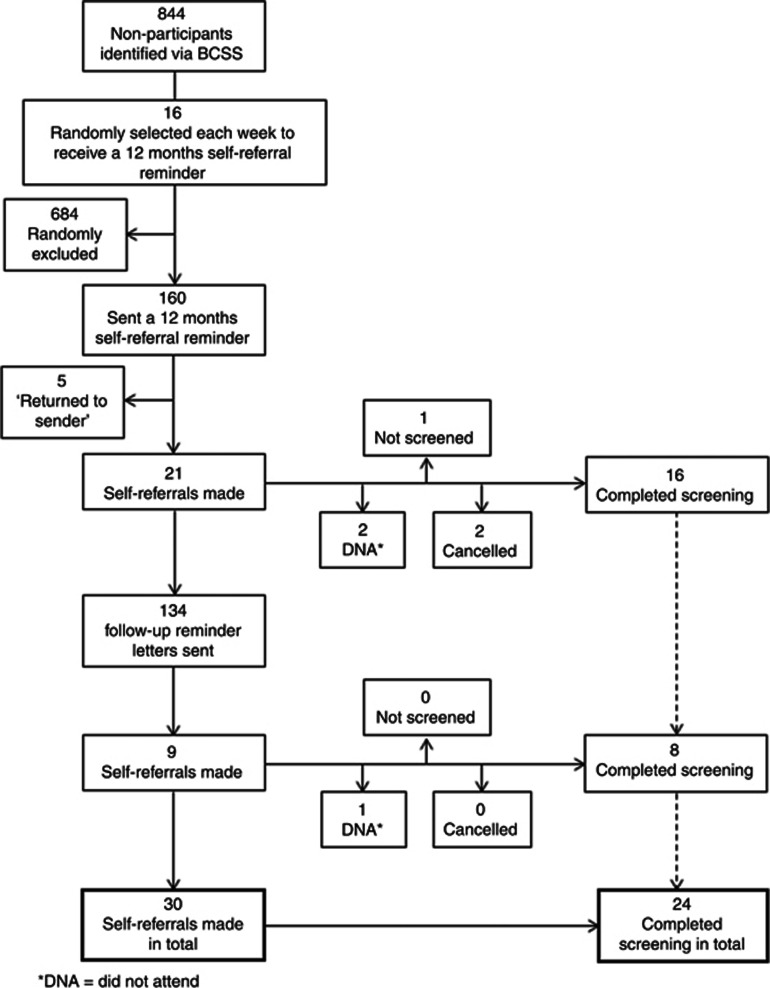
Basic design of the study.

A total of 160 people (male=71, 44.4% female=89, 55.6%) were randomly selected to receive a 12 months' self-referral reminder; however, five (3.1%) reminders were found to be undeliverable and were ‘returned to sender'. Subsequently, 155 people (male=68, 43.9%, female=87, 56.1%) were monitored as part of this study (Figure 2). Variation by locality and IMD score tertile are shown in Table 2.

### BSS-screening referrals and attendance

A total of 30 (19.4%) adults self-referred for BSS. Of these, 24 (80%) attended their appointment and were screened, 3 (10%) did not attend, 2 cancelled and 1 did attend but was not screened owing to high blood pressure (Figure 2). The overall attendance rate was therefore 15.5% (24/155).

Attendance differed significantly by gender, with more women attending an appointment than men (*n*=21 (20.7%) *vs n*=9 (8.8%), respectively; OR=2.73, 95% CI=1.02–7.35, *P*=0.05). There were no statistically significant differences between localities (Brent *vs* Harrow) or tertiles of area-level deprivation (Table 3).

The self-referral attendance rate of eligible adults not included in this feasibility trial during the study period was 1.2% (8/684).

### Process evaluation

#### Self-referral method

Of the 30 people who self-referred for a BSS appointment, 28 (93.3%) did so by returning the ‘appointment–request slip' the remaining two (6.7%) did so by calling the provided Freephone telephone number.

#### Follow-up reminder reminder

A total of 21 people (13.5%) responded to the self-referral reminder within 4 weeks. Subsequently, 134 follow-up reminders were sent with a further nine (6.7%) responses received within the remaining 8-week response period. No responses were received beyond the 12-week cut-off period.

#### Preference for day and time slot of appointment

Of the 30 people who self-referred for a BSS appointment, 24 (80%) expressed a preference for a specific day and/or time. It was not possible to accommodate preferences for 12 people (50%); only one went on to cancel.

#### Preference for gender of practitioner

Of the 30 people who self-referred for a BSS appointment, 27 (90%) indicated a preference for a same-sex practitioner; none (0%) indicated a preference for a practitioner of the opposite sex; and three (10%) gave no preference. It was not possible to accommodate the preference of eight (30%) people; however, no-one asked to be rescheduled. Preferences for the sex of the practitioner were examined by gender: women were significantly more likely to request a same-sex practitioner than men, with all of the women who self-referred for an appointment requesting a same-sex practitioner, compared with two-thirds of men (100% *vs* 67% *χ*^*2*^=7.78, *P*<0.05).

## Discussion

This feasibility study was initiated to test the format and potential efficacy of incorporating a mailed self-referral reminder and locally tailored information leaflet into the current BSS invitation process.

The reminder, when sent with the locally tailored information leaflet 1 year after the participants' initial invitation, facilitated uptake in 15.5% of recipients, thereby exceeding the threshold for further investigation in a RCT (*n*=24 *vs n*=3). The self-referral rate for individuals not sent a 12 months' reminder during the study period was higher than anticipated (1.2% *vs* 0.35%); however, our results would have exceeded the minimum level of efficacy even assuming this higher rate (A'Hern, 2001).

The finding that this intervention is feasible and has the potential to improve uptake by this group is highly important; if the findings of this study were replicated in a large RCT, then this simple intervention could have a considerable impact on uptake at St. Mark's Hospital. A self-referral rate of 15% among previous non-responders would equate to an increase in overall uptake of ∼9% (estimated by multiplying the proportion of adults not responding to the initial invitation (0.6) by the proportion of adults attending screening in response to the 12 months' reminder (0.15)). This would increase overall uptake at St. Mark's to almost 50%. If similar rates are observed in a multicentre study, the implementation of a 12 months' reminder in the national programme could have considerable public health benefits (Geurts *et al*, 2015). Furthermore, additional reminders, possibly at 24, 36 and 48 months', have the potential to increase overall uptake even further. Finally by a process of elimination such additional reminders would target the most deprived population and could ultimately reduce the socioeconomic gradient in screening attendance.

Our study found that women were more likely to attend in response to the reminder than men. The 12 months' reminder, therefore, has the potential to reduce the gender gap that has been observed in response to the first invitation (McGregor *et al*, 2015a). Previous research has indicated that for women particularly, the possibility of having a male endoscopist leading the procedure is a barrier to uptake (Menees *et al*, 2005), and so the option to allow participants to communicate a preference for the gender of the endoscopist is likely to have explicitly and directly addressed this barrier, thereby encouraging women to re-consider BSS attendance. In addition, it is also likely that the leaflet had a role in facilitating uptake in women specifically, given that it was designed to reduce barriers to FS, and women have been found to report more barriers to the test than men (Wardle *et al*, 2005).

Although the proportions requesting a same-sex practitioner between men and women were significantly different in our study, the number of men and women self-referring in this study was small (*n*=9 and *n*=21 respectively) and may not be representative of the proportions of men and women who would request a same-sex practitioner in the general population. For instance, the current study was set in an ethnically diverse area and so the impact of the gender preference option may have been all the more apparent here, as previous research has found the gender of the endoscopist to be a pertinent barrier for black and ethnic minority women (Varadarajulu *et al*, 2002). Future work should aim to explore individual ethnicity when examining preferences for a same-sex practitioner.

Previous research examining uptake of BSS in response to the initial invitation has identified a strong socioeconomic gradient in participation, with rates varying from 33% in the most deprived areas to 53% in the least deprived (McGregor *et al*, 2015a). In this study, we found no significant differences in participation between tertiles of area-level deprivation (McGregor *et al*, 2015a); however, it is important to note that the study was not designed to test for differences between tertiles of area-level deprivation, and so may have been underpowered to detect such differences. If the finding were reproduced in a larger trial, the intervention examined in this study may represent a potential strategy to reduce socioeconomic inequalities within the national programme.

The main limitation of this study is that the intervention used contained multiple components (including gender preference, appointment preference and a locally tailored information leaflet), and so without the appropriate control groups the contribution of each factor to the success of the reminder could not be teased apart. The next step would be to perform a RCT testing each of these components with appropriate controls.

One potential concern regarding the leaflet used in this study is that information within was not balanced. It was specifically designed to promote uptake by addressing the barriers and highlighting the benefits associated with the test. However, as the leaflet supplemented the existing information (i.e., the standard leaflet–which is balanced–, the consent form–which outlines the risks of the procedure– and the face to face counseling–during which the risks are discussed with a specialist screening practitioner and which the individual must undergo prior to the screening procedure–), the requirements of consent when making a screening decision were still met in accordance with the General Medical Council guidelines (General Medical Council, 2008). Nonetheless, to examine satisfaction with the reminder leaflet, we will use waiting room questionnaires and compare responses with individuals receiving the standard NHS leaflet in the subsequent RCT.

At last, it is important to note that in the UK, one in six people have a reading level below that expected of an 11-year old (Harding *et al*, 2012); therefore, the leaflet may still have been too difficult for some people to read. Reducing the readability of written information further may have benefits over and above those of the current interventions used within our study; however, for these adults written materials may not be the most suitable. Researchers seeking to reduce inequalities should focus on alternative channels of engaging these adults, such as through community outreach and telephone intervention.

## Conclusion

This study found that a locally tailored information leaflet and mailed reminder letter, with options for the day and time of the appointment and the gender of the practitioner performing the test, was feasible, efficacious and exceeded a minimum level of efficacy needed to merit further investigation in a RCT.

## Figures and Tables

**Table 1:** Characteristics of the reminder leaflet

	**Description**
**Motivational characteristics**	
Easy to read	The Flesch formula was used to assess the readability of the leaflet, which gave it a score of 68.7 (equivalent of a 13–15-year reading age). Pilot participants indicated that the leaflet was ‘the right length', not ‘too positive' and ‘included enough information to make a decision about screening'.
General Practitioner cancer lead endorsement	The BSS programme was endorsed by the local general practitioner cancer lead for Hillingdon. A photograph and quote of the GP were included: ‘I would urge anyone aged 55–59 to take this quick, potentially lifesaving, one-off test that significantly reduces your risk of getting bowel cancer'.
Provincial social norms message	The leaflet included a descriptive provincial social norms message: ‘About 270 people take up the Bowel Scope Screening test at St. Mark's Hospital every month'.
Effective communication of risk	The antecedents and consequences of bowel cancer were used to communicate risk and explain the preventative mechanisms of bowel scope screening: ‘Bowel cancer develops from polyps, which are small growths in your bowel. Most polyps are harmless, but some can turn into cancer if left untreated. By removing any polyps in your bowel during the test, bowel scope screening is a very effective way of reducing the chance that you will get bowel cancer in the future'.
Patient narratives	The leaflet included two patient narratives (one male, one female). Narratives have been associated with a reduction in the perceived impact of barriers and increased perceived risk of CRC (Dillard *et al*, 2010), increased intention to be screened (McGregor *et al*, 2015b) and improved attendance at colonoscopy screening (Jensen *et al*, 2014). Female narrative: ‘I must admit I was nervous, but the specialist nurse explained everything very clearly. It wasn't painful at all. I was told I had no polyps and given the all clear, which was a huge relief. My friend died from bowel cancer 5 years ago, so I was determined this wouldn't happen to me!' Male narrative: ‘The staff at St. Mark's Hospital were great. The doctor found a polyp, which he removed. I didn't feel a thing. The doctor explained that polyps often don't have any symptoms, so people don't always know if they have them. I'm glad they found the polyp before it had a chance to become something more serious'.
Reducing worry about pain, discomfort and embarrassment associated with the procedure	The leaflet was designed to reduce worry about pain, discomfort and embarrassment associated with the procedure. Statements addressing pain were based on patient reported outcomes from the UK FS pilot study: ‘The test is done in private and nearly everyone says it's not embarrassing' ‘Most people say they felt no pain, or only mild pain' (Robb *et al*, 2012).
**Practical characteristics**	
Map and local transport options	The leaflet was designed to address practical barriers to screening. A map of the area, and description of local transport links to the hospital was included to help patients plan their journey.
Instructions on how to make an appointment	Instructions on how to make an appointment by telephone referral were reiterated in the leaflet. Patients were also informed they could call the St. Mark's Freephone telephone number for further information about the test.

**Table 2:** Sample characteristics of the reminder population

	***n***	**%**
**Gender**		
Male	68	43.9
Female	87	56.1
**Area**		
Brent	76	49.0
Harrow	79	51.0
**Tertile of deprivation (IMD score)**		
Tertile 1 (least deprived)	31	20
Tertile 2	62	40
Tertile 3 (most deprived)	62	40

**Table 3:** Uptake following the reminders by gender, tertiles of the Index of Multiple Deprivation and location in the eligible sample (*n*=155)

**Comparisons**	***n*** **attended (%)**	**OR (95% CI)**	***P-*****value**
Overall (*n*=155)	24 (15.5)	—	—
**Gender**			
Male (*n*=68)	6 (8.8)	—	—
Female (*n*=87)	18 (20.7)	2.73 (1.02–7.35)	0.05
**Location**			
Harrow (*n*=79)	12 (15.2)	—	—
Brent (*n*=76)	12 (15.8)	1.10 (0.35–3.40)	0.87
**Tertile of deprivation**			
1 (*n*=32, least deprived)	4 (12.5)	—	—
2 (*n*=64)	11 (17.2)	1.46 (0.39–5.51)	0.57
3 (*n*=63, most deprived)	9 (14.3)	1.08 (0.22–5.37)	0.93
