# Supplementary figures and images for: Use of a 12 months' self-referral reminder to facilitate uptake of bowel scope (flexible sigmoidoscopy) screening in previous non-responders: a London-based feasibility study
Source: Br J Cancer. 2016 Mar 15;114(7):751–8. doi: 10.1038/bjc.2016.43 (PMC4984863; doi:10.1038/bjc.2016.43)

Supplementary Material: Leaflet


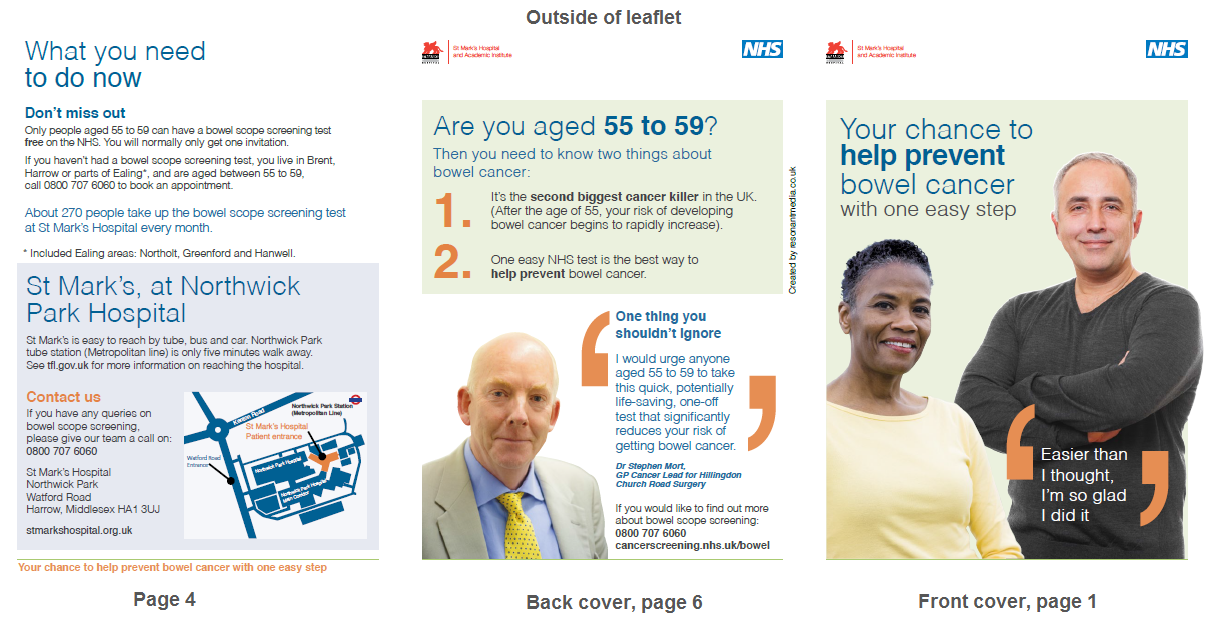


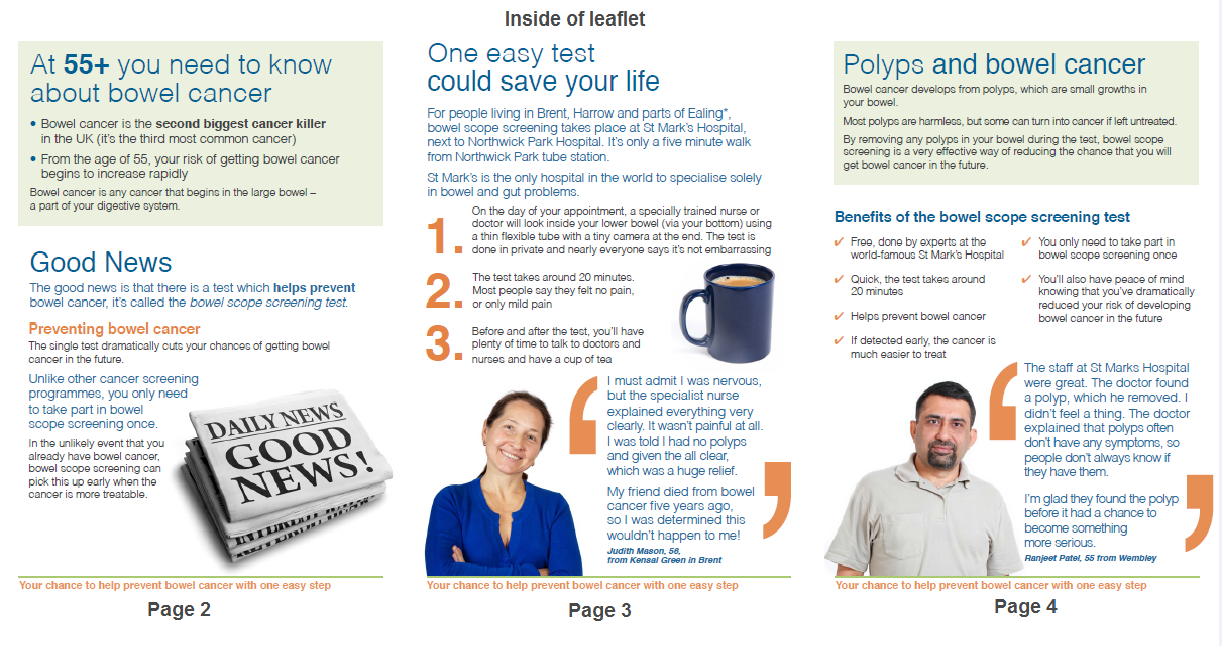

Supplement: Supplementary Material 1 [file bjc201643x1.doc]
