# Supplementary material for: Use of a 12 months' self-referral reminder to facilitate uptake of bowel scope (flexible sigmoidoscopy) screening in previous non-responders: a London-based feasibility study
Source: Br J Cancer. 2016 Mar 15;114(7):751–8. doi: 10.1038/bjc.2016.43 (PMC4984863; doi:10.1038/bjc.2016.43)
Supplement: Supplementary Material 3 [file bjc201643x3.doc]

St. Marks’ Bowel Cancer Screening Centre

St Mark’s Hospital

Watford Road

Harrow

Middlesex

HA1 3UJ

**Freephone Helpline** **0800 707 6060**

**<dd> <Month> <Year>**

<Title> <First Name> <Last Name>

<Address Line 1>

<Address Line 2>

<Address Line 3>

<Address Line 4>

<Postcode>

HA9 6HH

Dear <Title> <First Name> <Initial> <Last Name>,

**NHS No**: <NHS Number>

**Reminder: Please book your appointment - Important information about your health**

We recently wrote to invite you for bowel scope screening, a new test available only in England which **helps prevent bowel cancer.**

**Saving Lives**

**People aged 55+ are most at risk of getting bowel cancer, this test helps to prevent it. This test is for people who don’t have any signs of bowel cancer.**

You are being invited because people who are aged 55 and over are the most at risk of developing bowel cancer. Having a Bowel Scope Screening test between the age of 55 & 59 helps prevent you from getting bowel cancer in the future. This is an important test highly recommended for **everyone** who is **55-59** years of age.

**What you need to do**

Please read the enclosed leaflet, which gives more information about the test, and also has stories from people who have already been to St Mark’s for bowel scope screening.

To book your test, simply fill in and post back the form overleaf in the Freepost envelope provided (you don’t need a stamp). We will then arrange a date and time for your bowel scope screening appointment. It takes place locally at St Mark’s Hospital. Alternatively, you can **Freephone 0800 707 60 60 to book an appointment**.

If you have any questions, please call the St Mark’s Bowel Cancer Screening Centre on 020 8869 3543.

Yours sincerely,

**Sarah Marshall**

**Clinical Programme Manager, Bowel Scope Screening**

IMPORTANT – PLEASE CHECK YOUR DETAILS AND RETURN IN THE FREEPOST ENVELOPE

**Name:** <Title> <First Name> <Last Name>

**NHS Number:** <NHS Number>

**Post Code:** <Postcode>

**Please fill in your details** (either your home telephone number or your mobile number is required; this is so we can contact you to confirm your appointment)**:**

Home number:

Mobile number:

Please **tick** this box if you would like to have a bowel scope screening appointment:

- I’d like to arrange a bowel scope screening appointment at St Mark’s Hospital in Harrow.

Please **tick** your preference:

- I would prefer to have a **Male** practitioner to perform my test.
- I would prefer to have a **Female** practitioner to perform my test.

Please **tick** as appropriate: My preferred appointment time(s) would be:

|  | **Afternoon**  **13:00-15:30** | **Evening**  **16:45-19:00** |
| --- | --- | --- |
| **Monday** |  |  |
| **Tuesday** |  |  |
| **Wednesday** |  |  |
| **Thursday** |  |  |
| **Friday** |  |  |

When we receive your form, we’ll contact you with a suggested date and time for your appointment.

**(Tear here)**
